# Protein—protein binding supersites

**DOI:** 10.1371/journal.pcbi.1006704

**Published:** 2019-01-07

**Authors:** Raji Viswanathan, Eduardo Fajardo, Gabriel Steinberg, Matthew Haller, Andras Fiser

**Affiliations:** 1 Department of Chemistry, Yeshiva University, New York, NY, United States of America; 2 Departments of Systems & Computational Biology, and Biochemistry, Albert Einstein College of Medicine, Bronx, NY, United States of America; Icahn School of Medicine at Mount Sinai, UNITED STATES

## Abstract

The lack of a deep understanding of how proteins interact remains an important roadblock in advancing efforts to identify binding partners and uncover the corresponding regulatory mechanisms of the functions they mediate. Understanding protein-protein interactions is also essential for designing specific chemical modifications to develop new reagents and therapeutics. We explored the hypothesis of whether protein interaction sites serve as generic biding sites for non-cognate protein ligands, just as it has been observed for small-molecule-binding sites in the past. Using extensive computational docking experiments on a test set of 241 protein complexes, we found that indeed there is a strong preference for non-cognate ligands to bind to the cognate binding site of a receptor. This observation appears to be robust to variations in docking programs, types of non-cognate protein probes, sizes of binding patches, relative sizes of binding patches and full-length proteins, and the exploration of obligate and non-obligate complexes. The accuracy of the docking scoring function appears to play a role in defining the correct site. The frequency of interaction of unrelated probes recognizing the binding interface was utilized in a simple prediction algorithm that showed accuracy competitive with other state of the art methods.

## Author summary

Protein–protein interactions are key to understand the molecular level mechanisms of regulation in the cell. However, there is still a limited understanding of what distinguishes a protein-protein binding site from the rest of the surface. This lack of knowledge is manifested in the relatively low accuracy of computational methods that try to predict protein interfaces. In this work we report a new conceptual insight about protein interfaces. Our results suggest that protein interfaces serve as generic binding sites to any ligand. This also means that in the absence of the known binding partner it is still possible to define protein interfaces by extensive docking studies of randomly selected, unrelated ligands, as they have a strong tendency to bind to the cognate binding site. This insight was leveraged in a new binding interface prediction algorithm that alone outperforms state of the art approaches that often combine a variety of features.

## Introduction

Specific protein-protein interactions are essential for maintaining a robust phenotype. A deeper understanding of these interactions would allow the identification of cognate ligands[1] and drivers of specificity, opening a pathway to manipulating the corresponding interaction interfaces in drug design applications[2]. While it has been estimated that a protein on average interacts with 3–10 other proteins[3], the Protein Data Bank[4] (PDB) contains a disproportionally small fraction of known protein complexes. For most of the PDB entries neither the ligand protein nor the protein binding interfaces are known. In response to this important problem, a number of methods have been developed to predict protein binding interfaces using structural information, which may be available in the form of known experimental or computational three dimensional models[5]. The methods to predict protein interfaces can be grouped into two main approaches: (1) homology-based and (2) *ab initio*. Homology-based predictions of interfaces rely on the knowledge of known protein complexes to infer the likely binding sites in similar proteins. These methods can be very powerful[6, 7], but their applicability is limited by the amount of known interfaces. Within the category of “*ab initio*” protein interface predictions a number of studies have attempted to identify distinctive features of interfaces[8–14] often employing various machine learning approaches. These features include residue composition[15], residue conservation[16–18], hydrophobicity[19, 20], planarity[15], predicted secondary structural features[14, 21], electrostatics[22], accessible surface area, among others.

Some studies found that different subtypes of protein interfaces (e.g. transient interfaces, interfaces between homo- and heteromers, etc.) have distinct sequence features, which can be exploited to predict some of the interface residues from sequence[14, 23]. For example, these features suggest that interfaces for obligate complexes are somewhat more hydrophobic and larger than other interfaces[15, 24]. Similarly, binding site hot-spots have been predicted using residue composition, conservation analysis, or other structural features such as desolvation effects[13]. However, a generic conclusion after many studies and using larger and more diverse test sets is that protein interfaces do not have a specific composition or other universal features they share[18, 25, 26]. This is arguably the expected conceptual conclusion as it is difficult to conceive a universal external evolutionary pressure that would unify interfaces[27]. Current success rates for protein binding interface predictions on a residue level are just barely statistically significant when compared to random predictions[28].

Relevant to the current study are the works that discuss the possible generality of binding site locations, both for small molecule and protein ligands. In the case of the former, it has been observed as early as in the 1980s that small organic molecules, both substrates and non-substrates tend to bind to similar, energetically favored “sticky” sites irrespective of their relevance to the target. These observations were made by experimental studies that soaked target proteins in organic solvents and examined the crystal[29] or NMR[30] structure for invariable small molecules sticking to energetically favorable sites. Computational methods such as the GRID[31], or the Multicopy Simultaneous Search (MCSS)[32] approach, as well as some of the most competitive methods currently available[33], are also based broadly on this observation.

It was observed in the late 1990s that protein superfolds (frequently occurring proteins that share their overall structural topology but have a range of distinct functions) have “supersites”. In other words, despite substantial sequence divergence and the evolved distinct functions, the 10–15 superfolds that dominate about half of the structural fold population of the genomes[34] usually have very similar binding site locations[35]. This observation was subsequently revisited and expanded to remote homologs with insignificant sequence similarity to the cognate ligands for a range of different fold topologies[36, 37].

Docking programs have been used successfully to predict partner-specific interface residues such as the Atomic Contact Frequency (ACF)[38] or the Residue Contact Frequency (RCF) method[39] and others[40]. These approaches require the prior knowledge of the cognate ligand from other, indirect sources, such as high throughput screening methods.

In the current work, we explored the generality of the phenomenon of binding supersites. We report the surprising observation that protein-protein interaction sites serve as generic protein binding sites. Protein ligands, irrespective of their relevance to the receptor protein, tend to bind to the cognate protein interface. This behavior does not depend on the docking program used, the range and type of protein ligand probes employed, or more technical conditions such as the size of the binding sites considered. Based on this new observation we introduce a docking-based, *ab initio* method for binding site prediction that does not require prior knowledge of the cognate ligand. Binding interfaces are determined by the frequency of a receptor residue interacting with a range of unrelated protein ligands in extensive docking simulations. A conceptual insight brought to light by our work is that protein shapes evolved to allow a surprisingly small number of suitable surface patches for interactions that are apparently sampled by a wide range of possible ligands. Alternatively, it may be that a variety of unique residue patterns that evolved for recognizing a specific cognate protein ligand also present an energetically relatively favorable site for non-cognate proteins.

## Results and discussion

### Unrelated protein ligands bind preferentially to the same receptor binding site

**Fig 1:**
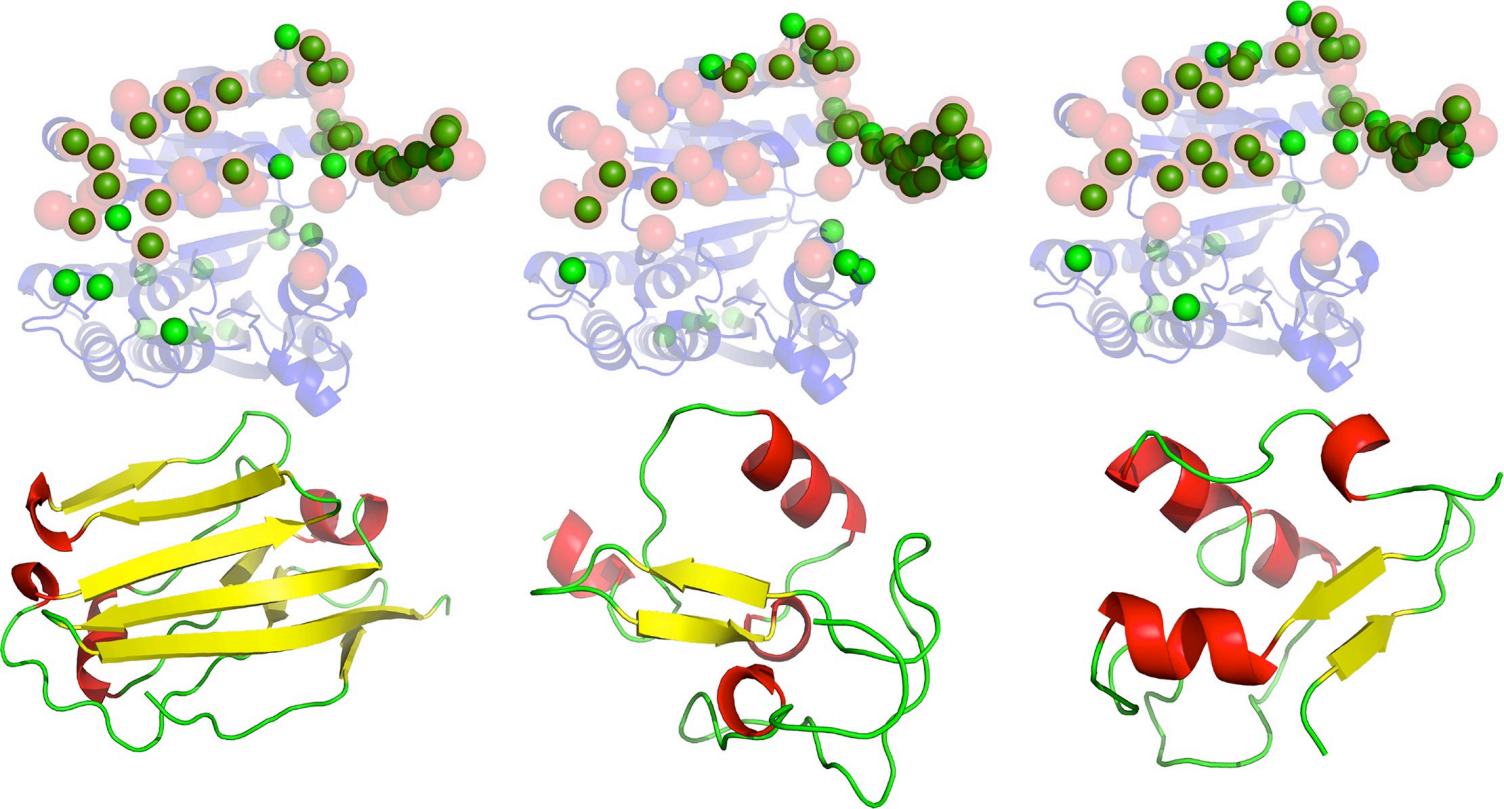
Binding supersite of 1cnz.A. Three non-cognate ligands (lower row, from left to right, PDB codes: 2jjs.C, 2v86.A, 3h33.A) that share no detectable sequence or structure similarity to the cognate ligand, are docked extensively on the surface of the receptor (upper row, 1cnz.A). In the upper row, ribbon model in transparent blue shows the receptor structure. The annotated functional site in the receptor is shown using red transparent spheres for the C_α_ atoms. The predicted functional site residues, as defined by the corresponding ligand probes underneath, is shown using green spheres for the C_α_ atoms.

We explored the hypothesis of whether protein-protein interaction sites also serve as generic binding sites for a range of non-cognate ligands, and as such, behave similarly to protein-small-molecule-binding sites[30, 32, 41, 42]. This would qualitatively generalize the observations made about supersites in superfolds[35]. We explored the preferred binding sites for a set of unrelated ligands on a large set of receptor proteins. Surprisingly, we found that unrelated ligands have a strong tendency to dock to the same general area of a receptor as its cognate ligand. We illustrate this in Fig 1, where three, topologically different ligands (all beta– 2jjs.C; mixed alpha and beta– 3h33.A; and a small protein fold with few secondary structures– 2v86.A), sharing no detectable structural or sequential similarity to the cognate ligand, all have a strong tendency to dock to the cognate protein binding site on the receptor protein (1cnz—3-Isopropylmalate dehydrogenase from Salmonella typhirium).

We explored the overall phenomenon by docking 13 different ligand probes, six immunoglobulin folds and seven randomly picked small protein folds on a combined target dataset of 241[43, 44] proteins with structurally defined protein binding sites. We ranked the residues in the receptor protein based on the RIF score (see Methods). The statistical significance of the agreement of the top ranked residues and the cognate binding site was assessed by using hypergeometric distribution to model the probability of correctly selecting an interface residue by chance. Out of the 241 target proteins, in 157 ±2 cases (or 65.2 ± 0.9%) the binding site was docked by a variety of unrelated ligands in a statistically significant manner. We evaluated the performance by randomly selecting 2000 models from the total set of 26000 docked models (13 X 2000 per ligand probe) and calculated the average performance and the standard deviation.

**Fig 2:**
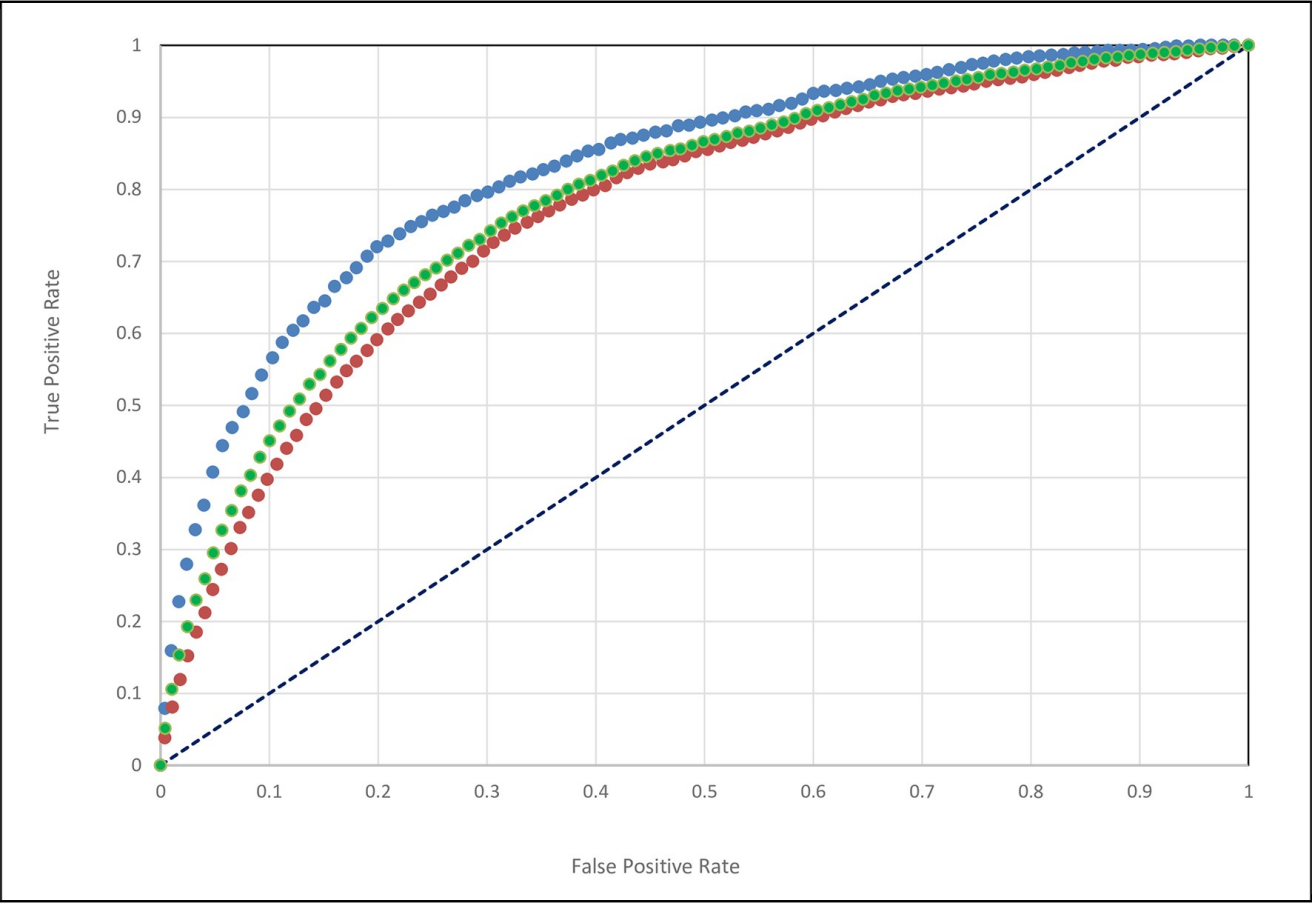
ROC curve for the combined set of 241 test proteins; blue: Docking Benchmark dataset; orange: NOX dataset; green: combined data set. AUC values: DOCKB = 0.83; NOX = 0.77; Combined = 0.79.

We further broke down results by complex and database type. Performance on the Docking Benchmark[44] and NOX[43] databases were 70.3% ± 1.1 and 61.1 ± 1.6, respectively. Furthermore, the NOX database contained a relatively well-balanced set of obligate(73) and non-obligate complexes(60), and the results on these subsets were 68.7 ±1.9 and 51.8 ±2.2, respectively. We also evaluated the results using sensitivity/specificity ROC curves (Fig 2.) and obtained an Area Under the Curve value of AUC = 0.79 for the combined set, while 0.83 and 0.77 for the Docking Benchmark and NOX databases, respectively.

All these suggest that the observation about protein binding supersites is a generic feature of proteins, with some fluctuation of specific success rates depending on the choice of test database.

**Fig 3:**
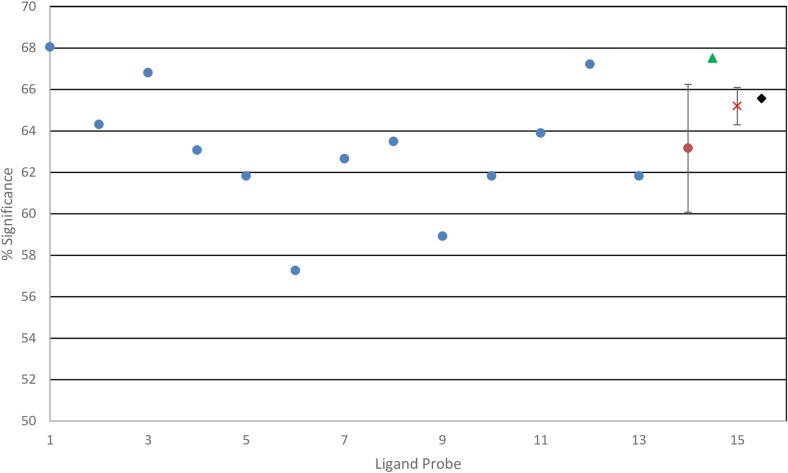
Performance dependence on probe. Dependence of % Significance on the choice of ligand probes using ZDOCK. The performance of each of the 13 probes is shown separately on the combined set of 241 query proteins. The overall average of these independent 13 performances is shown in red X with standard deviation. Orange circle with standard deviation shows the average result of 2000 structures drawn randomly from the 26,000 total docked structures pooled from the 13 probes. Green triangle shows the performance of the 13 single cognate ligands, one for each of the query proteins. Black square indicates performance if all docked poses from all probes are used together.

We also explored how well the cognate ligands bind to and define the annotated functional site of the receptor proteins in comparison to unrelated ligands. (Fig 3) Interestingly, while the cognate ligands have a tendency to better recognize the interface, this tendency is statistically not significantly different from the results obtained for unrelated ligands.

### Effects of choice of ligand probes, receptor size and docking programs on the accuracy of results

We further subdivided our results as a function of different ligand probes and ligand sizes, while also exploring two alternative docking programs, ZDOCK and GRAMM, to examine the role that variations in the scoring functions play in detecting supersites. We found little dependence on the type of probe used with either docking program (Fig 3). The differences in results obtained using individual probes are mostly statistically insignificant. The success rate for the NOX dataset depending on the ligand probes ranged between 54.1 to 65.4% with an average success rate of 60.1% ± 3.9% using ZDOCK, while the success rate ranged from 36.8% to 51.9% with an average of 44.7% ± 4.4% using GRAMM. ZDOCK appears to yield slightly better results with the immunoglobulin superfamily probes, while GRAMM works better with the non-immunoglobulin set of ligand probes. If we use a consensus prediction from all 13 ligand probes, the performances in the case of ZDOCK and GRAMM are 60.1% and 44.7%, respectively. The better performance of ZDOCK suggests that the energy function may play a role in defining the “stickiness” of protein binding supersites. ZDOCK[45] uses a statistical pair potential with a limited set of amino acid residue types, while the GRAMM[46] energy function is arguably more general using a step function that includes a classic repulsion term.

We compared the actual interface residues predicted by the two docking programs, ZDOCK and GRAMM. Although the entire set of interface residues predicted by the two docking programs were not identical, for 40% and 79% of the 241 proteins in the data set, the two docking programs predicted more than 10 or more than 5 interface residues in common out of 15, respectively. To put these numbers in a statistical context: the expected number of residues that are common out of 15 residues between any two random draws,—in protein sizes 100, 150, 200, 250 and 300 are: 2.27, 1.58, 1.16, 0.82, and 0.81 residues, respectively. Consequently, the two programs have a strong tendency to locate binding sites similarly. The corresponding p-values of observed common residues between ZDOCK and GRAMM are all significant at any protein size.

**Fig 4:**
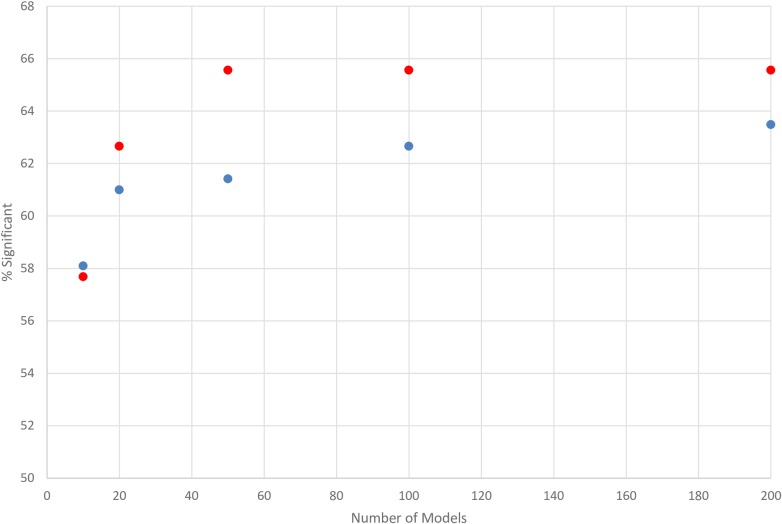
Effect of % significance on the number of models used (first 200 docked models (red) or the last 200 docked models (blue) as ranked by the scoring method of ZDOCK.

We explored an additional aspect of the potential impact of the employed energy function. ZDOCK ranks the generated docked poses by their energy score, so we explored if there is a difference in performance between the top-scoring and bottom-scoring docked poses. Indeed, this phenomenon can be observed once we plot the performance of the first and last 200 docked poses (Fig 4). There is a weak but persistent tendency that energetically higher ranked poses are more useful in identifying binding sites (Fig 4). These small differences disappear as the number of sampled conformations approach 200 and beyond. The differences between the accuracy of ZDOCK and GRAMM and between top-ranked and bottom-ranked docked poses of ZDOCK suggest that a more accurate energy function will identify binding sites more accurately because the relative affinity of non-cognate ligands will be better captured.

**Fig 5:**
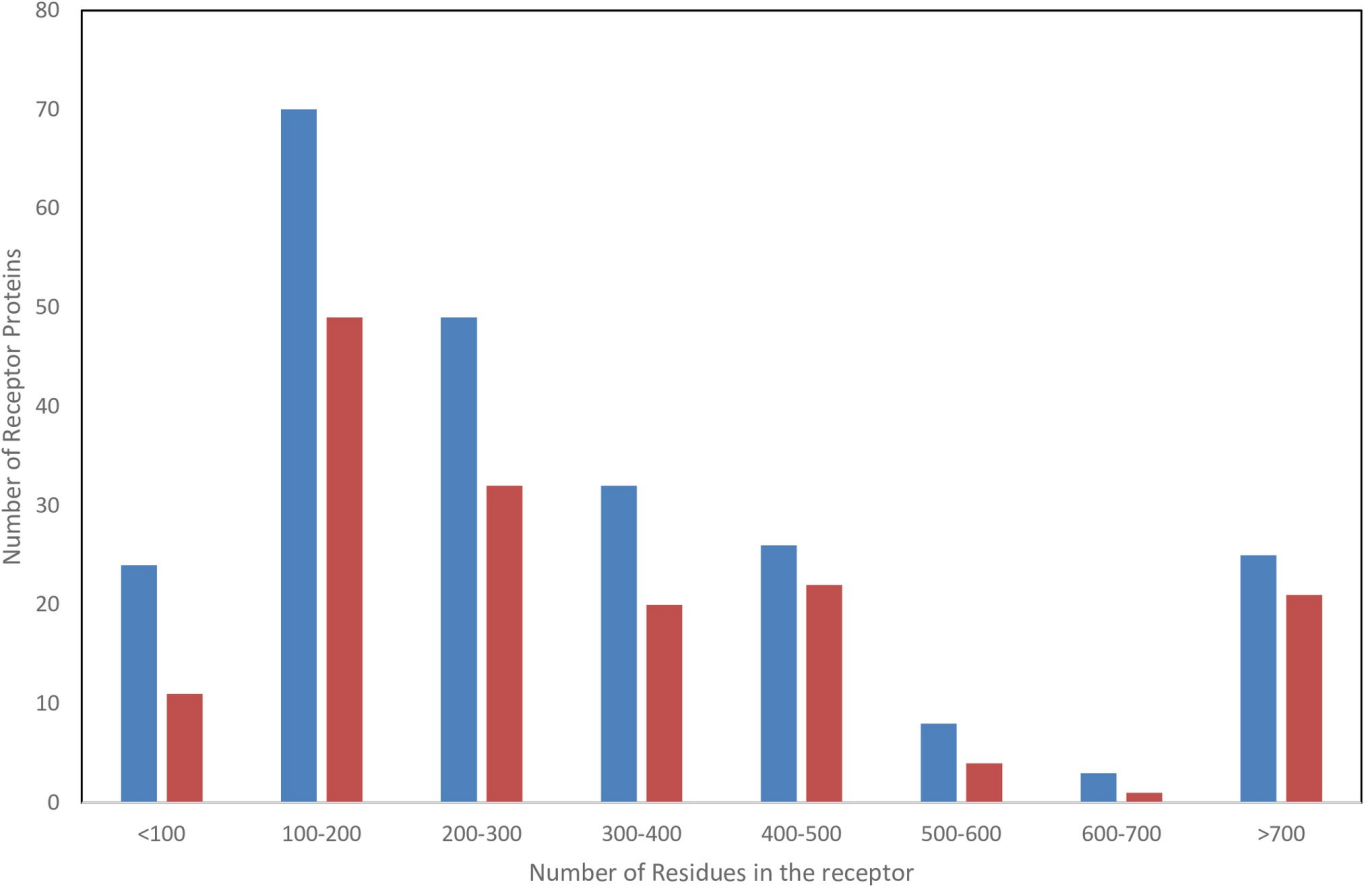
Number of receptor proteins (blue) in each size range and the number of successful predictions (orange) in each range.

When considering the possible reasons for the existence of protein binding supersites, besides the general energetic preferences of certain “sticky” areas of the protein, one could also consider receptor-shape-driven causes. For instance, one could speculate that in the case of small proteins it might be a geometrical artifact that only a confined area is suitable to accept interactions. However, the distribution of the size of receptors in the current work has a large range (<100 residues to >700 residues) for which the ability to detect supersites appears to be uniformly high (Fig 5).

### Effect of using different subsets of docked complexes

We further dissected the possible differences in performance between the two docking approaches. First, we compared the performance of these techniques using 2000 models generated by the methods, irrespective of the size of the identified binding interface, with the performance when using only a subset of the docked complexes that have the most common interface sizes; in the current work, formed by 9 residues (Table 1). Though the GRAMM docking method appears to sample a larger fraction of all the residues in the protein (85.9% vs 72.1%) as well as the interface residues (99.6% vs 97.5%), ZDOCK identifies a larger number of true interface residues ranking in the top 15 positions (60.1% +/- 3.9 for ZDOCK vs. 44.7% +/- 4.4 for GRAMM). In case of considering 9–residue patches only, as expected, the total number of residues sampled (40.7% for ZDOCK and 54.6% for GRAMM) as well as the interface residues sampled (39.6% for ZDOCK and 76.8%) is smaller, which apparently has a strong influence on the method performance. In particular, the GRAMM docking method performs significantly worse when a subset of docked complexes, consisting only 9-residues is used in the analysis with a % significance of 21.6 ±3.4 compared to 48.4 ±4.7 using ZDOCK.

**Table 1:** Sampling of residues by the different docking methods, within the entire protein and on the interface only, using all docked complexes, and using only a subset of docked complexes where the interface is made of 9 residues.

Docking Method	% total residues sampled	% Interface residues sampled	% total residues sampled in 9-residue patches	% Interface residues sampled in 9-residue patches
ZDOCK	72.1	97.5	40.7	39.6
GRAMM	85.9	99.6	54.6	76.8

### The effect on accuracy of the number of docked conformations and number of probes

**Fig 6:**
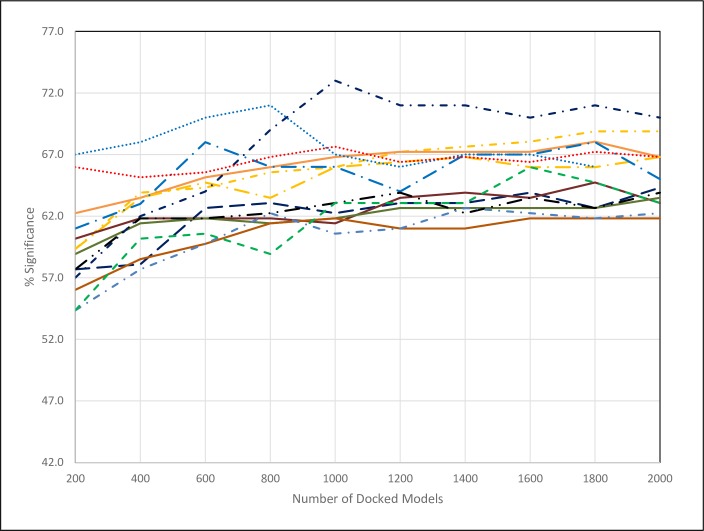
Dependence of % significance as a function of the number of docked models used for each ligand probe. The red dotted line represents the average of all the 13 probes. All other lines are for the individual probes.

We used 13 different ligand probes and by default 2000 docked conformations to locate the binding site of a receptor protein. This amounts to 13x2000 = 26,000 docked poses. We gradually reduced the number of docked poses and found that with 13 ligands as few as 200 docked conformations are sufficient to establish the same results as before, with 2000 poses (Fig 6).

Another aspect of the binding site exploration is the number and variety of probes employed. Upon plotting the performance of all the 13 probes independently, it is clear that these perform in a relatively tight range and that the observed small differences most likely can be acknowledged to the particular set of test proteins used. As an empirical test, the accuracy using ZDOCK changes from 65.4 when averaged over a subset of 6 randomly selected different probes to 63.2 when averaged over all 13 different probes. We found that randomly selecting 2–3 probes already provides robustly the same performance results as running all 13 probes (Fig 6).

### Effect of using uncomplexed target proteins

It has been shown that docking based methods are less successful to predict the correct binding pose and binding site when targeting uncomplexed receptors, especially the ones that undergo substantial conformational change upon binding to their cognate ligand. In our case we do not restrict our analysis to the cognate ligand and to a few (or one) docked poses with the lowest energetics, as such an approach is likely to be insensitive to small conformational changes. Non-cognate ligands bind with much lower affinity, and we are capturing the relative preference of any ligand to dock to the cognate binding site. We manually identified 95 target proteins in our combined set for which we could locate a PDB structure in an uncomplexed form. The F-scores of "apo" and "holo" forms for this subset of 95 target proteins is include in the Supplementary Information (S1 Fig). On this subset, the success rate of capturing binding sites has an average F-score of 0.27 and 0.26 for the complexed and uncomplexed targets, respectively, a statistically insignificant difference.

### Performance within and outside of superfolds

An important aspect of this study is to explore if the observed phenomenon is a function of fold types, or something more general. The distribution of protein folds is very uneven[34], with 12 superfolds populating about one third of the human genome. It has been discussed in the literature that these superfolds have a tendency to preserve their ancient/general binding interface despite their divergence into a range of distinct functions[35]. We analyzed our dataset to examine whether the well-performing interface detections using unrelated ligand probes work disproportionally well for these superfolds. Of the 241 protein chains, 91 belong to one of the top 12 CATH[47] superfamilies, roughly recapitulating the proportion of superfolds in biological systems. The success rates for the 91 superfamily and 131 non-superfamily classified cases are 71.0% and 62.2% using ZDOCK, respectively, and then 30.0 and 40.8 using GRAMM (Table 2). These small and non-systematic differences suggest that there is no preference for superfamily proteins, and that supersites are characteristic to all protein folds. Further breaking down of the results in a benchmark database dependent fashion shows that the general performance on the Docking Benchmark dataset is significantly better with ZDOCK than with the GRAMM docking approach, and for the NOX dataset these differences are substantially reduced (Table 3). However, no systematic preference emerged of supersites in superfolds, in fact, non-superfold subsets outperform in two out of four subsets (ZDOCK with NOX database, and GRAMM with Docking Benchmark).

**Table 2:** Performance of identifying binding sites as a function of superfamily classification on the combined set of 241 test proteins.

CATH Superfamily	Total # of cases	Significance (ZDOCK) (%)	Significance (GRAMM) (%)
Yes	91	71.0	30.0
No	131	62.6	40.8
MIixed	17	82.3	43.8
Not Classified	2	50.0	50.0

**Table 3:** Performance for DOCKB and NOX datasets.

CATH	DOCKB Dataset			NOX Dataset		
Superfold Classification	Total #	% Success (ZDOCK)	% Success (GRAMM)	Total #	% Success (ZDOCK)	% Success (GRAMM)
Yes	50	80.0	16.3	41	61.0	46.3
No	56	60.7	45.4	75	64.0	37.3
Mixed	2	100.0	0.0	15	80.0	46.7
Not Classified	0			2	50.0	50.0

In order to understand some of the differences in performances, we examined the specific superfamily classifications of the proteins represented in the two datasets (Table 4). In the Docking Benchmark, we found a highly skewed distribution of superfolds, where 66% of the superfamily classification is “immunoglobulin-like” while 14% are classified as the Rossman fold. Meanwhile, the NOX dataset is slightly better balanced, with the Rossman, TIM-barrel, and Immunoglobulin-like folds comprising 48.8%, 19.5%, and 17% of the dataset, respectively. It is possible that ZDOCK is better tuned to dock immunoglobulin like folds and their over-representation has shifted the results higher in the Docking Benchmark dataset.

**Table 4:** Performance for individual superfold members.

CATH Superfamily	DOCKB Dataset			NOX Dataset		
	Total #	% Significant		Total #	% Significant	
		(ZDOCK)	GRAMM		ZDOCK	GRAMM
Rossman Fold	7	28.6	14.3	20	50.0	50.0
Immunoglobulin Like	33	84.8	6.3	7	85.7	71.4
TIM Barrel	1	100.0	0.0	8	75.0	37.5
Four Helix	-	-	-	2	50.0	50.0
Trefoil, Acidic Fibroblast growth factor	1	100.0	100.0	2	100.0	0.0
alpha-beta plaits	1	100.0	0.0	-	-	-
OB Fold	3	100.0	33.3	1	0.0	100.0
Jelly roll	3	100.0	33.3	-	-	-
Globin like	1	100.0	0.0	-	-	-
Alpha-beta barrel	-	-	-	1	100.0	0

### Comparison to other interface prediction methods

Slightly different interface definitions can drastically change the number of residues involved in the interface. A recent study suggests that even in the case of nearly identical definitions, the disagreement between different definitions can be substantial, suggesting that a ~0.8 F-score as a practical upper limit for prediction methods[48]. In addition, residues not involved in direct contact with a ligand can have a profound effect on binding, as illustrated by a number of studies[2]. Meanwhile, random predictions are distributed with a peak around 0.1 F-score[28] but many individual random predictions reach up as high as 0.2 F-score. Current protein interface prediction methods that provide results on a residue level and with an F-score accuracy, report statistically significant but generally speaking fairly low accuracies[28, 49, 50]. For instance, Table 3 in Taherzadeh et al.[49] published this year, reports seven methods, with F-score performances in the range of 0.18–0.31. These methods typically use different benchmark datasets therefore a substantial part of the variation among the performance probably can be acknowledged to that fact. To put our results in this general context we converted our performance into F-score evaluation and obtained an average F-score of 0.35 using ZDOCK and 0.22 using GRAMM, which compares well with the recent values in the literature using other methods to identify protein-protein interfaces. The good performance is especially promising as our approach is based on the direct evaluation of a single feature while all other methods are using a combination of a number of features in machine learning setting.

### Conclusion

In this work, we have shown that protein binding supersites exist in proteins, i.e. the protein binding interface provides an energetically-preferred binding site for many alternative, non-cognate proteins as well. There were previous, anecdotal studies that noted that even non-cognate ligand have tendency to accumulate around the cognate site, as it was shown in case of chymotrypsin when docked with a non-native binder, lysosyme[40]. Other recent studies also pointed in the direction of our current observation[51, 52]. Employing an energy landscape based analysis it was observed that binding sites can be identified without the prior knowledge of the cognate ligand. In that study, in a strict filtering protocol, the few lowest energy binders were identified for subsequent mapping of their preferred binding poses. Though this approach delivered an effective prediction method, it left open the following question—are these low energy binding poses related to the cognate binding partner, and thereby representing similar binding affinities, and likely, a similar binding interface? Also, the observations were not generalized, the successful cases were not analyzed in terms of protein topology, to illustrate if the observations go beyond the original observations made about superfolds, where binding sites are preserved despite a long evolutionary history of sequence divergence. We observe that these sites can be effectively detected by employing an extensive docking sampling with a range of unrelated protein ligand probes. In another study the Hex docking approach was used in cross docking experiment and suggested the existence of “favored” sites[53]. The authors have noted a tendency of these sites to be closer to the center of mass of the protein and explored residue type preferences of binding patches. A wide variety of probes were used with different topologies but the phenomenon was not generalized in terms of distribution on folds, to see if these observations are generic over all fold types or work mostly for superfolds as it was established in 1998[54]. The accuracy of this approach to detect protein binding sites is comparable to other state-of-the-art techniques. However, it uses a mostly orthogonal input in comparison to many existing technologies, and as such, a practical outcome of this study is both a new, standalone binding site prediction algorithm and an opportunity to improve existing binding site predictions by incorporating this information with other existing techniques that use residue preferences, conservation, geometrical definitions, among others. On the conceptual level, our observations argue that possibly a combination of geometrical restraints (shape of the local molecular surface) and energetically preferred residue patterns are responsible for establishing these supersites. Given past experience and our current results, we believe that the number of combinations of how an energetically “sticky” patch can be established varies substantially. However, the fact that docking algorithms, which combine shape complementarity with a scoring function that assesses interactions, are able to capture many of these sites suggests a path forward in the characterization of protein interfaces. Docking methods were benchmarked in a number of studies that showed a lack of strong correlation between calculated and experimental binding affinities[55]. The current study implicitly confirms this observation when we show that the success of identifying binding interfaces does not depend in a statistically significant manner on whether one uses cognate or non-cognate ligands, albeit a small trend favoring cognate ligands can be detected. This suggests that more generic energetic features are captured.

## Materials and methods

### Datasets and definition of interface

Two different datasets were employed in this study. A set of 108 protein chains from the Docking Benchmark[44] and another set of 133 protein chains from the NOX database[43], 73 and 60 of which are obligate and non-obligate complexes, respectively. The protein binding interfaces were identified from the three dimensional structure of the complexes using the CSU[56] program. A residue was considered to be at the interface if any of its atoms is within 3.5 Å of any atom of the interacting protein in the complex and establishes a legitimate contact type according to the CSU classification.

### Interface prediction method

In our approach we use a total of 13 ligand probes, none of which are known partners or share any detectable sequence similarity to known ligands for the query proteins in our data set. Six of these ligand probes were immunoglobulin folds (PDB[57] codes: 1i85.D, 2jjs.C, 2wbw.C, 1t0p.B, 2ptt.B, 3udw.C), as we assumed this fold evolved to be particularly suitable and generic to explore protein surfaces. Seven others were selected randomly. PDB entries were split into chains and clustered at 25% sequence identity level. All protein solved by NMR and not within the range of 70–250 residues were removed. From the remaining set we selected 7 proteins (between 70–120 residues) with different topologies compared to one another (1whz.A, 2eaq.A, 2v86.A, 2w8x.A, 2y2y.A, 3h33.A, 5cuk.A).

Two different docking programs, ZDOCK[45] and GRAMM[58] were used to generate a maximum of 2000 docked complexes for each of the protein chains in our dataset with each of the 13 ligand probes. The 2000 complex structures for each receptor-ligand probe pair were analyzed using CSU to identify the residues at the interface, R_ik_, where *i* is the residue position number and *k* is the *k*th docked complex structure. If a residue is at the interface, then I(R_ik_) = 1; otherwise, I(R_ik_) = 0. A Residue Interface Frequency (RIF), N_i_ was determined for each residue at position *i* in the receptor protein by summing over all the 2000 docked structures.

$$N_{i}=\sum_{k=1}^{2000}{I(R}_{ik})$$

The residues were then ranked based on the N_i_ values, and the top 15 ranking residues were considered most likely to be at the interface. The residues were also ranked similarly by using a subset of the 2000 complex structures all of which contained exactly nine residues at the interface (the most frequent interface patch size during the simulations). This subset generally consisted of between 150 and 300 complex structures.

### Performance evaluation

The actual number of interface residues varies with each receptor protein. We considered the number of true positive predictions of interface residues in the top 15 rankings assigned by our method. The performance of the current RIF method was evaluated using a statistical significance test by comparing it with a random prediction. The probability of randomly selecting x interface residues in the top K predicted residues (K = 15 in our case) for a query protein chain with N is the total number of residues sampled during the extensive docking simulation and M actual interface residues is given by the probability mass function of the hypergeometric distribution:

$$P(X=x)=\left(\begin{array}{c} M\\ x \end{array}\right)\left(\begin{array}{c} N-M\\ K-x \end{array}\right)/(\begin{array}{l} N\\ K \end{array})$$

An interface prediction is considered significant if P(X = x) < 0.05. The performance is expressed as

$$\%\mathrm{significance}=\frac{Number\;with\;P(X=x)<0.05}{Total\;number\;in\;the\;dataset}X\;100$$

Theoretically, the hypergeometric distribution can be exposed to some instability when very small numbers of discrete residues are assessed for significance; therefore, performance was also evaluated empirically, by randomly sampling 15 residues from the surface exposed residues sampled during the extensive docking simulation of the query protein 200 times and finding the average number of interface residues, μ, and the standard deviation, σ. A Z-score was then calculated, Z = (N–μ)/ σ, where N is the actual number of interface residues in the top 15 using the RIF method. The prediction was considered significant if Z > 1.97. The % significance evaluated using the hypergeometric distribution and the random sampling method yielded identical results.

Receiver operating characteristic curves ROC were calculated by plotting the true positive rate (sensitivity) against the false positive rate (1- specificity). Corresponding Area Under the Curve values were obtained.

Functional residues are a small fraction of the total residues, so true negatives far outnumber true positives. Therefore methods that heavily reward true negatives, such as the “specificity” and the “accuracy”, are less appropriate than ones that do not, such as the “F-Score”[59] and appropriately F-scores were used in a number of previous studies. Therefore success of a functional residue prediction was also assessed by the F-score, the harmonic mean of precision and recall (2*precision*recall / (precision + recall)), where precision is the ratio of true positives to the sum of true and false positives and recall is the ratio of true positives to the sum of true positives and false negatives.

## Supporting information

S1 Fig Head-to-head comparison of F-scores of the interface prediction method of apo and holo proteins for a set of 95 proteins chosen from the Dockb and NOX datasets. (TIF)

S1 Text List of complexed-uncomplexed structures used in Fig 1. (TIF)
