# Supplementary figures and images for: Protein—protein binding supersites
Source: PLoS Comput Biol. 2019 Jan 7;15(1):e1006704. doi: 10.1371/journal.pcbi.1006704 (PMC6336348; doi:10.1371/journal.pcbi.1006704)

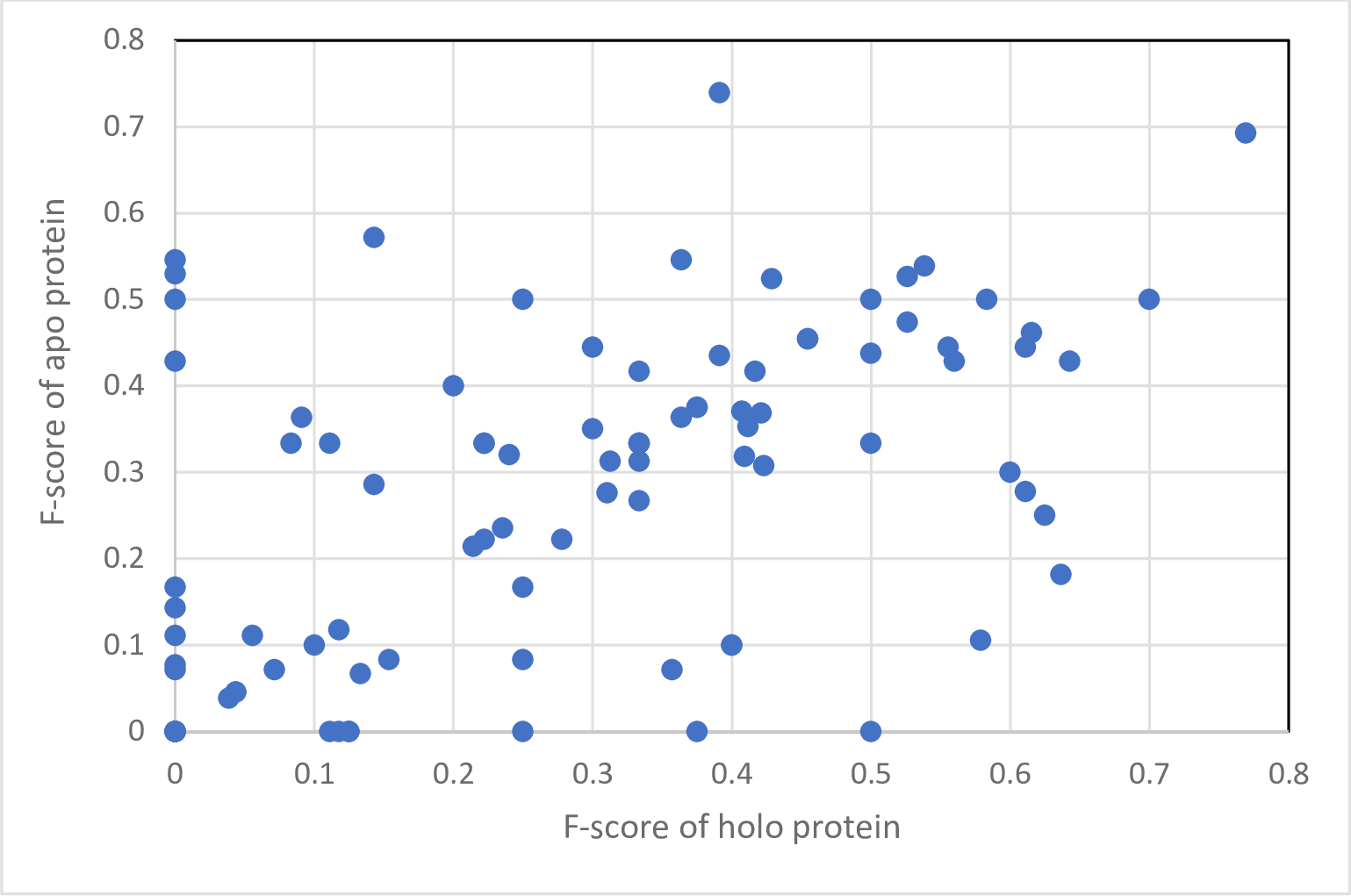

Supplement: S1 Fig — (TIF) [file pcbi.1006704.s001.tif]

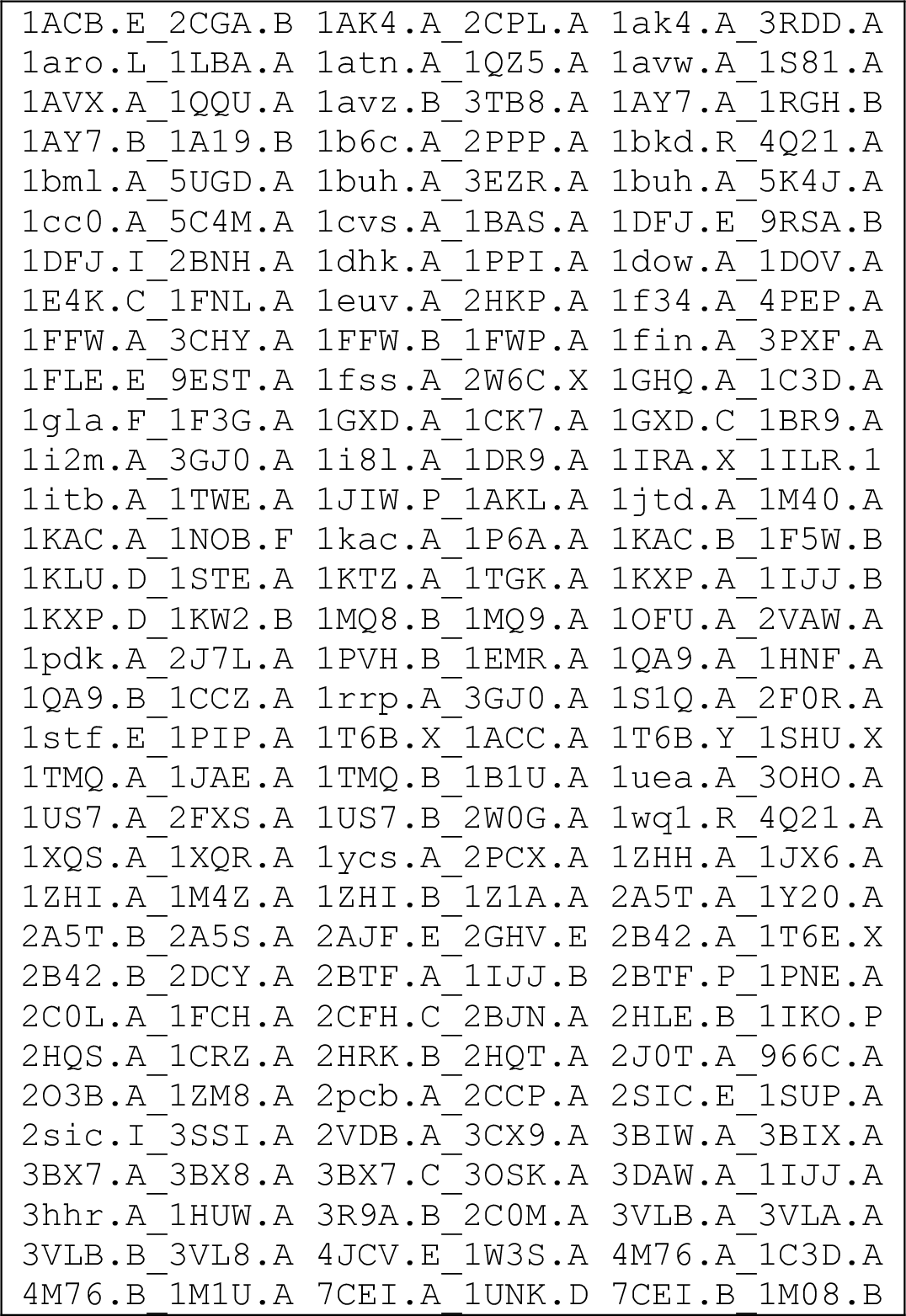

Supplement: S1 Text — (TIF) [file pcbi.1006704.s002.tif]
